# Is There a Relationship between Lead Exposure and Aggressive Behavior in Shooters?

**DOI:** 10.3390/ijerph15071427

**Published:** 2018-07-06

**Authors:** Nisha Naicker, Pieter de Jager, Shan Naidoo, Angela Mathee

**Affiliations:** 1 Environment & Health Research Unit, South African Medical Research Council, P.O. Box 87373, Houghton, Johannesburg 2041, South Africa; amathee@mrc.ac.za; 2 The Epidemiology and Surveillance Section, National Institute for Occupational Health, National Health Laboratory Services, 25 Hospital St, Constitution Hill, Johannesburg 2000, South Africa; 3 School of Public Health, Faculty of Health Sciences, University of the Witwatersrand, Private Bag 3, Wits, Johannesburg 2050, South Africa; Pieter.deJager@wits.ac.za (P.d.J.); shan.naidoo@wits.ac.za (S.N.); 4 Environmental Health Department, Faculty of Health Sciences, University of Johannesburg, Johannesburg 2028, South Africa; 5 Department of Anaesthesia, School of Clinical Medicine, Faculty of Health Sciences, University of the Witwatersrand, Johannesburg 2193, South Africa

**Keywords:** shooting ranges, aggression, Buss-Perry Aggression Questionnaire, blood lead levels, South Africa

## Abstract

Lead exposure has been associated with psycho-neurological disorders. Elevated blood lead levels have been found in shooters. This study assesses the association between the blood lead levels of shooters and their levels of aggression. An analytical cross-sectional study was conducted in Gauteng, South Africa. Participants were recruited from four randomly selected shooting ranges with three randomly selected archery ranges used as a comparison group. A total of 118 (87 shooters and 31 archers) participants were included in the analysis. Aggressiveness was measured using the Buss-Perry Aggression Questionnaire. Shooters had significantly higher blood lead levels (BLL) compared to archers with 79.8% of shooters versus 22.6% of archers found to have a BLL ≥ 5 μg/dL (*p* < 0.001). Aggression scores were significantly higher in shooters (*p* < 0.05) except for verbal aggression. In the bivariate and regression analyses, shooters with BLLs ≥ 10 μg/dL were significantly associated with the hostility sub-scale (*p* = 0.03, adjusted odds ratio (OR) 2.83, 95% confidence interval (CI) 1.103–7.261). Shooters have a significantly higher BLL and aggressiveness compared to archers. However, elevated blood lead levels were significantly associated with hostility only. Interventions need to be put in place to prevent continued exposure and routine screening of populations at risk should be implemented.

## 1. Introduction

Since the 1970s, firearms and shooting ranges have been recognized as high risk for elevated lead exposure [1,2]. Mean blood lead levels (BLL) observed in studies of users of shooting ranges have ranged from 10 µg/dL to over 40 µg/dL [3,4,5]. Several studies have shown that the BLL may take a substantial amount of time to decrease after the cessation of shooting especially in those with very high levels or those that have practiced shooting for extended periods [6,7,8,9]. Higher BLLs were associated with a larger caliber of firearm [10], higher frequency of shooting [3,6], and the use of indoor rather than outdoor shooting ranges [3]. Whether the bullet was jacketed or not did not significantly impact BLL [11].

Ingestion and inhalation are the primary routes of lead exposure in shooting ranges [12,13]. Inhalation occurs when the gun is fired and fine airborne particles from the primer are released [14]. Both fine and coarse particles may contaminate hands, skin, hair, clothing, shoes, and other surfaces and could potentially be ingested especially in case of poor hand hygiene [3,15]. It is also possible that lead particles may be transported to other environments including vehicles and homes.

Lead exposure contributed to approximately 16.8 million disability-adjusted life years (DALY’s) to the global burden of disease in 2013 [16,17]. Even though lead primarily targets the nervous system [13,18], it is a multifaceted toxic substance with systemic clinical manifestations involving the cardiovascular [19,20], renal [21,22], hepatic [23], reproductive system [24,25], and immune system [26]. Lead exposure has been associated with psycho-neurological disorders such as behavioral changes, impaired cognitive function in adults, and reduced intelligence in children [13,18,27,28]. Aggressive behavior in adulthood following childhood lead exposure has been documented in several studies [29,30]. However, there is a relative dearth of information on lead exposure in adult shooters especially in developing countries and its association with aggressive behavior. In this study, we examined the association between BLL in users of shooting ranges and aggressive behavior.

## 2. Materials and Methods

### 2.1. Study Population and Sample

An analytical cross-sectional study was conducted at randomly selected shooting and archery ranges in Johannesburg, Pretoria, and Ekurhuleni in Gauteng Province, South Africa. A total of 17 archery ranges associated with the South African National Archery Association and 64 South African Police Service accredited shooting ranges were identified for possible inclusion. Nine archery ranges and 26 shooting ranges were randomly selected and invited to participate in the study. Three archery ranges (response rate 33%) and four shooting ranges (response rate 15%) gave written, informed consent and agreed to participate in the study.

The total population of shooters and archers was not available. Therefore, the sample size of 384 was calculated for an unknown population size to achieve a 95% confidence interval and a power of 0.9 using a sample size calculator. Participants were only included in the study if they were over the age of 18 years, were not pregnant, provided signed, informed consent, and were regular shooters (they were not merely attending to obtain a firearm license). There was no randomization of individual participants and all shooters meeting these criteria were included. Similarly, all archers were invited to participate in the study.

The total number of people interviewed was 121. However, three participants also worked at the shooting range and were excluded from the study. The final sample consisted of 87 gun shooters and 31 participants that were exclusively archers. Fifteen participants in the gun shooters group were also archers. Those who are regular gun shooters were included in the gun shooter group.

### 2.2. Data Collection

BLLs were measured using the LeadCare^®^ II Blood Lead Testing System immediately or within 24 h of collection. The system required 50 μL of whole blood, which were obtained from a finger prick after participants washed their hands with soap and water. The lower limit of detection for the LeadCare^®^ II Blood Lead Testing System was 2 µg/dL and the higher limit was 60 µg/dL. All biological specimen collections and testing was performed by a registered medical practitioner. Participants with a blood lead level of more than 25 µg/dL were referred for venous blood collection and testing of blood lead levels in an accredited laboratory. All participants found to have a blood lead level of greater than 25 µg/dL were counseled and referred to their preferred medical practitioner for further investigation. Participants were also provided with information on the risks associated with lead exposure and practical steps, which can be taken to reduce exposure to lead.

A self-administered questionnaire was completed by each participant. The questionnaire collected information on socio-economic factors, details on shooting and hygiene behaviors, current health status, and an assessment of aggressive tendencies using the Buss Perry Aggression Questionnaire [31]. This questionnaire has four sub scales: Physical Aggression, Anger, Hostility, and Verbal Aggression. Physical aggression consisted of nine questions and the possible scoring ranged from 9 to 45. Anger and hostility consisted of 8 items with a scoring range of 8 to 40. Verbal aggression had 5 items and scoring ranged from 5 to 25. The Buss-Perry Aggression Questionnaire has been widely utilized and validated to investigate aggressiveness in various populations [32,33,34].

### 2.3. Data Analyses

Data were captured in Excel and the data set was cleaned and inspected for errors before being exported to Stata/SE version 14.2, which was used to conduct the analyses. Sex, education, and other categorical data are described in terms of frequencies, proportions, and percentages. Continuous data such as the Buss-Perry aggression scores (total score, verbal aggression, physical aggression, hostility, and anger) were presented in terms of measures of spread and central tendency. The spearman rank order correlation matrix was done to assess the correlation between BLL and aggression scores. Univariate analysis for individual characteristics and blood lead level outcome variables of interest were compared using χ^2^ for categorical data. The Mann-Whitney U test was utilized in the univariate analysis of nonparametric continuous variables (Blood lead levels and aggression scores). The level of significance was taken at *p* ≤ 0.05. Blood lead levels, which are the independent variables in the regression analysis, were categorized as <10 µg/dL and ≥10 µg/dL. The action level of 5 µg/dL based on the revised Centers for Disease Control and Prevention guidelines and the NIOSH Adult Blood Lead Epidemiology and Surveillance (ABLES) program, which were applicable during the time the study was conducted, was included in the bivariate analyses [35]. Buss Perry aggression scores were categorized above and below the geometric mean for each sub scale. Independent variables were included in the regression model if the *p* value was ≤0.05 and crude and adjusted OR’s were produced.

### 2.4. Ethics

Ethical Approval for the study was obtained from the University of Witwatersrand Human Research Ethics Committee (Medical), clearance certificate number: M130251.

## 3. Results

### 3.1. Characteristics of Shooters and Archers

Socio-demographic factors such as age, sex, household income, and employment were the same between archers and shooters. However, there was a significant difference (*p* value < 0.05) in tertiary education. A total of 84% of archers have tertiary education compared to 54% of shooters.

Blood lead levels in the total sample (*n* = 118) ranged from 2.0 μg/dL to 60.0 μg/dL with a median blood lead level of 7.1 μg/dL. The median blood level in archers was 2.7 μg/dL (range from 2 μg/dL to 7.1 μg/dL). Shooters had a median BLL of 11.9 μg/dL (range from 2.0 μg/dL to 60.0 μg/dL). Among shooters, 80% had a BLL ≥ 5 μg/dL while 23% of archers had the same BLL. Approximately 40% of shooters and 3% of archers had a BLL ≥ 10 μg/dL. There was a significant difference in the blood lead levels between shooters and archers (*p* value < 0.001). The most important risk factor for elevated BLL among shooters was the frequency of shooting. Those shooting weekly or more frequently had the highest BLL (*p* value < 0.001) (Table 1).

### 3.2. Aggression Scores

For the physical aggression, anger, hostility sub scales, and total aggression scores were assessed through the Buss-Perry scale. Shooters scored significantly higher when compared to archers (Table 2). However, the verbal aggression subscale was similar between the groups. The geometric mean is 7.1 for shooters compared to 6.1 for archers (*p* = 0.30).

A spearman’s correlation was run to assess the relationship between blood lead levels and aggression scores in shooters. There was a weak yet positive significant correlation between blood lead levels and anger scores, r_s_ = 0.225, *p* < 0.05. Physical aggression, hostility, and verbal aggression were positively correlated but not significant (Table 3).

The bivariate analyses were conducted for risk factors of aggressiveness in shooters (Table 4). In shooters, the only factor significantly associated with all subscales of aggression was the frequency of shooting. Participants that practiced shooting one or more times a week were significantly more likely to report aggressiveness (physical, verbal aggression, hostility, and anger). Employment, income, and education did not have an effect on aggression. Blood lead levels ≥ 5 µg/dL were not associated with aggression. However, shooters with BLL ≥ 10 µg/dL had a significant association with hostility (*p* value < 0.01, Crude OR’s 2.47, 95% CI 1.014–6.019). Post adjustment for shooting frequency, the OR was 2.83 (*p* value 0.03, 95% CI 1.103–7.261).

## 4. Discussion

This study has shown that blood lead levels ≥ 5 µg/dL and aggression scores are significantly higher in study participants who used shooting ranges compared to those using archery ranges. Frequency of use of shooting ranges was significantly associated with higher blood lead levels as well as aggression scores in shooters. Multiple studies have shown an association between shooting and high blood lead levels [3,5,36,37]. Erle et al. (2017) conducted a study assessing aggression in target shooters at shooting clubs compared to basketball players [38]. The study showed that target shooters self-reported more aggression and this increased over time compared to basketball players. Target shooters also had greater aggressiveness and more negative thoughts that occurred after target shooting [38]. A meta-analytic review assessing the effects of weapons on aggressiveness concluded that exposure to weapons such as guns increases aggressive thought, hostility, and aggression [39].

Based on the authors’ knowledge, this is the first study investigating the association between aggression and elevated BLLs in an adult (≥18 years old) population in shooting ranges. All factors were weakly yet positively correlated with BLLs. However, only anger was significantly correlated. In the regression analyses, the findings show that hostility scores were significantly elevated if lead exposure levels were ≥10 µg/dL. However, the associations with the other sub-scales (Physical aggression, Verbal aggression, and Anger) were not statistically significant in this study. Aggression and anti-social behavior in adolescence [40,41,42] and children [43,44,45] have been associated with elevated lead levels and lead exposure early in childhood. Ecological studies have pointed to an association between early childhood exposure and crime rates [46,47]. Therefore, despite the findings from this study, the relationship between lead exposure and aggressive behavior in adults should be studied further.

There are several limitations to this study. The cross-sectional design does not allow for the establishment of causal relationships between the utilization of shooting ranges, BLL, and levels of aggression. Cross-sectional studies also do not allow the determination of the relationship directionality such as whether aggressive individuals have a preference for shooting or whether potential lead exposure associated with the use of shooting ranges predisposes shooters to aggression. The causes of aggressive behavior are multifactorial and include factors such as education levels, gender, violent family environments, substance abuse, interaction with delinquent peers, and victimization [48]. In this study, information on a broad range of factors associated with aggression was not collected and the small sample size was inadequate to control for additional factors. However, there is strong evidence from the literature to corroborate the role of lead in aggressive behavior [32,33,34]. Participants were not sampled randomly. Therefore, there is the potential for selection bias in this study since more aggressive and distrusting shooters may have been less inclined to participate. In addition, vulnerable populations such as women (*n* = 7) and shooters with occupations involving the use of firearms (such as policemen or shooting instructors) were not adequately represented in the study. In addition, the LeadCare^®^ II Blood Lead Testing System validated in previous research has a detection limit of 2 µg/dL and 60 µg/dL. Therefore, its use is limited when compared to Inductively Coupled Plasma Mass Spectrometry (ICP-MS), which is the gold standard for blood lead measurements. However, for the purposes of this study, participants with high blood lead levels of ≥60 µg/dL had venous blood sent to an accredited laboratory for confirmation. This study assessed acute lead exposure. However, many shooters may have been chronically exposed to lead from shooting ranges. Studies have provided evidence of the detrimental impact of chronic low level lead exposure on the neurological system. Therefore, a longitudinal study assessing lead exposure and behavior will add further value.

In this study, the population was restricted to adults (over the age of 18 years). However, it was observed during fieldwork that a considerable proportion of children also visited shooting ranges with their fathers/parents. The implications for their lead exposure (as well as that of women and shooting range workers who were under-represented in this sample) and health outcomes require further investigation.

## 5. Conclusions

This study has highlighted the association between shooting range use and lead exposure. In addition, there is a significant link to aggressive behaviour among shooting range users in this setting when compared to archers. However, the extent to which lead exposure contributed to increased aggression scores is not clear in this study. Only the hostility subscale was significantly associated with blood lead levels ≥10 µg/dL. Interventions such as the use of lead-free ammunition, proper ventilation, and hygiene practices as well as regular inspections of shooting ranges and blood lead testing of recreational and occupational shooters should still be considered.

## Figures and Tables

**Table 1:** Characteristics of Shooters and Archers.

Factor	Total*n* = 118 (%)	Shooters*n* = 87 (%)	Archers*n* = 31 (%)
**Age**			
Median	36	38 years	32 years
Range	18–74	18–74 years	19–64 years
**Sex**			
Female	17 (14.4)	9 (10.3)	8 (25.8)
Male	101 (85.6)	78 (89.7)	23 (74.2)
**Income**			
<R10000	23 (19.4)	17 (19.5)	6 (19.3)
≥R10000	81 (68.6)	57 (65.5)	24 (77.4)
**Education**			
No Tertiary	41 (34.7)	36 (41.3)	5 (16.1) **
Tertiary Education	72 (61.0)	46 (54.0)	26 (83.9)
**Employment**			
Employed	107 (90.7)	79 (90.8)	28 (90.3)
Not Employed	9 (7.6)	6 (6.9)	3 (9.7)
**Frequency of Shooting**			
Monthly - <Weekly		55 (63.2)	
1 or More Times a Week		29 (33.3)	
**Usual Duration of Shooting**			
≤1 h		14 (16.1)	
>1 h		72 (82.8)	
**Blood Lead Levels**			
Mean	9.3	11.5	2.9
Geometric Mean	6.2	8.5	2.7
Range	2–60	2–60	2–7.1
<5 µg/dL	41 (34.7)	17 (20.2)	24 (77.4)
≥5 µg/dL	74 (62.7)	67 (79.8)	7 (22.6) **
≥10 µg/dL	36 (30.5)	35 (40.2)	1 (3.2) **

** *p* value < 0.001.

**Table 2:** Aggression scores of shooters and archers.

Variables	Shooters (*n* = 87)			Archers (*n* = 31)			*p* Value
	Geometric Mean	Mean (sd)	Range	Geometric Mean	Mean (sd)	Range	
Physical Aggression	16.8	17.2 (3.9)	12–34	14.8	15.0 (2.8)	12–25	0.001 **
Anger	14.8	15.1 (3.6)	10–27	13.1	13.2 (1.9)	10–17	0.01 *
Hostility	14.4	15 (4.6)	8–30	12.3	12.6 (2.9)	8–21	0.01 *
Verbal Aggression	7.1	7.6 (3.3)	5–24	6.4	6.6 (1.8)	5–11	0.30
Total Score	53.4	54.7 (13.0)	37–111	46.8	47.3 (7.3)	37–72	0.002 *

* *p* value < 0.01, ** *p* value < 0.001 (Mann Whitney U test/Wilcoxin Rank-Sum).

**Table 3:** Spearman rank order correlation matrix for BLL and aggression scores in shooters.

Variable	BLL	Physical Aggression	Anger	Hostility	Verbal Aggression	Total Aggression Score
BLL	1					
Physical Aggression	0.077	1				
Anger	0.225 *	0.432 **	1			
Hostility	0.206	0.583 **	0.458 **	1		
Verbal Aggression	0.094	0.708 **	0.379 **	0.565 **	1	
Total Aggression Score	0.213	0.814 **	0.728 **	0.837 **	0.755 **	1

* *p* value ≤ 0.05. ** *p* value ≤ 0.001.

**Table 4:** Factors affecting aggression scores in shooters.

Factor	N (%)	Physical Aggression	Anger	Hostility	Verbal Aggression	Aggression
		Mean, *p* Value	Mean, *p* Value	Mean, *p* Value	Mean, *p* Value	Mean, *p* Value
**Sex**						
Female	9 (10.3)	16.8	15	15.4	8.3	56
Male	78 (89.7)	16.9, 0.96	15.2, 0.90	14.9, 0.76	7.5, 0.50	54.5, 0.75
**Income**						
<R10000	19 (21.8)	17.9	15.4	15.7	8.3	56.7
≥R10000	55 (63.2)	17.2, 0.53	15.3, 0.96	15.3, 0.79	7.5, 0.39	55.1, 0.67
**Education**						
No Tertiary	36 (41.3)	17.8	15.5	15.3	7.6	55.1
Tertiary Education	46 (52.9)	16.9, 0.37	14.9, 0.47	15.1, 0.85	7.8, 0.82	55.0, 0.98
**Employment**						
Employed	79 (90.8)	17.2	15.2	15.1	7.6	54.7
Not Employed	6 (6.9)	17.2, 0.96	14.2, 0.51	14.7, 0.83	7.8, 0.86	53.8, 0.88
**Frequency of Shooting**						
Monthly - <Weekly	55 (63.2)	16.3	14.5	13.5	6.4	5.7
1 or More Times a Week	29 (33.3)	18.9, 0.004 **	16.4, 0.02 *	17.4, 0.0002 **	9.7, <0.0001 **	62.6, 0.0001 **
**Usual Duration of Shooting**						
≤1 h	45 (51.7)	17.3	15.0	14.8	7.3	53.9
>1 h	41 (47.1)	17.1, 0.83	15.1, 0.92	15.3, 0.61	7.9, 0.34	55.6, 0.58
**Blood Lead Levels (BLL)**						
<10 µg/dL	51 (58.6)	16.9	15	13.9	7.3	53.1
≥10 µg/dL	36 (41.4)	17.4, 0.61	15.3, 0.67	16.5, 0.009 *	8.1, 0.23	56.8, 0.21
<5 µg/dL	18 (20.7)	16.6	14	14.6	8	52.1
≥5 µg/dL	69 (79.3)	17.3, 0.54	15.4, 0.15	15.1, 0.73	7.5, 0.61	55.4, 0.37

* *p* value <0.05; ** *p* value <0.001.
